# Pb speciation and elemental distribution in leeks by micro X-ray fluorescence and X-ray absorption near-edge structure

**DOI:** 10.1107/S1600577523006616

**Published:** 2023-08-24

**Authors:** Jianling Sun, Yongqiang Yang, Liqiang Luo

**Affiliations:** a Beijing Municipal Research Institute of Eco-Environmental Protection, Beijing 100037, People’s Republic of China; b National Research Center for Geoanalysis, Beijing 100037, People’s Republic of China; c National Engineering Research Centre for Urban Environmental Pollution Control, Beijing 100037, People’s Republic of China; Bhabha Atomic Research Centre, India

**Keywords:** heavy metal, spatial distribution, synchrotron radiation, XANES, detoxification mechanism

## Abstract

Vegetables are crucial to a human diet as they supply the body with essential vitamins, minerals, *etc*. Heavy metals that accumulate in plants consequently enter the food chain and endanger people’s health. Studying the spatial distribution and chemical forms of elements in plant/vegetable tissues is vital to comprehending the potential interactions between elements and detoxification mechanisms. In this study, leek plants and soil from vegetable gardens near lead–zinc mines were collected and cultivated with 500 mg L^−1^ PbNO_3_ solutions for three weeks. Micro X-ray fluorescence was used to map the distribution of Pb and other chemical elements in leek roots, and X-ray absorption near-edge spectroscopy was used to assess the Pb speciation in leek roots and leaves. These findings demonstrated that Pb, Cu, Mn, Cr, Ti and Fe were detected in the outer rings of the root’s cross section, and high-intensity points were observed in the epidermis. Zn, K and Ca, on the other hand, were distributed throughout the root’s cross section. Leek root and leaf contained significant quantities of lead phosphate and basic lead carbonate at more than 80%, followed by lead sulfide (19%) and lead stearate (11.1%). The capacity of leek roots to convert ambient lead into precipitated lead and fix it on the root epidermis and other inner surfaces is a key mechanism for reducing the toxic effects of Pb.

## Abstract 2

Leek roots can physiologically convert about 80% of environmental lead into insoluble lead phosphate and lead carbonate, and leek leaves also contain similar proportions of lead phosphate and lead carbonate. An essential mechanism of Pb detoxification is the development of significant amounts of lead precipitation and immobilization on the root epidermis and other inner surfaces.

## 1. Introduction

Vegetables are a major source of vitamins, minerals and fiber, all of which are necessary for a healthy diet and have protective antioxidant effects on human tissue (Ali & Al-Qahtani, 2012 ▸; Islam *et al.*, 2016 ▸). Growing in heavy-metal-contaminated soil or irrigating with heavy-metal-rich sewage will result in the accumulation of heavy metals (HMs) in both edible and inedible parts of the plant (Rahman *et al.*, 2013 ▸). The HMs in vegetables subsequently enter the human body through dietary intake, causing several clinical and physiological problems (Khan *et al.*, 2008 ▸; Sharma *et al.*, 2007 ▸). Lead (Pb), one element that we are interested in and have been studying for many years, is extensively distributed in the environment and can impair the human neurological, immunological and vascular systems and the kidneys (Garg & Aggarwal, 2011 ▸; Päivöke, 2003 ▸). Meanwhile, excessive Pb in plants can also lead to growth retardation, chloro­sis and root blackening by altering membrane structure and permeability, disrupting enzyme activity, blocking mitosis and distorting chloro­plast structure (Li *et al.*, 2012 ▸; Sharma & Dubey, 2005 ▸). Pb is the most toxic trace metal for plants with a toxicity threshold below 1 µ*M*, with only mercury being nearly as toxic as Pb (Kopittke *et al.*, 2010 ▸). Compared with other HMs, such as Cu, Zn and Cr, Pb in vegetables is higher than national standards, and has caused serious pollution (Zhao *et al.*, 2016 ▸).

Leafy vegetables are more likely to introduce HMs into humans than other vegetables, as HMs tend to accumulate in their edible parts (Fan *et al.*, 2017 ▸; Zhao *et al.*, 2016 ▸). Crops such as cauliflower, kale and cabbage have been reported to absorb more metals without causing significant toxicity symptoms to plants (Zayed & Terry, 2003 ▸; Zayed *et al.*, 1998 ▸). Atmospheric fallout of ultrafine particles enriched with metals can also be transferred directly through leaves (Mombo *et al.*, 2016 ▸). Therefore, the greater impact of leafy vegetable contamination on the human food chain is also of concern, and is especially true in areas with heavy metals sources, such as near mining areas. Understanding the accumulation characteristics of HMs and formulating effective measures to prevent HMs from entering the soil–vegetable system is necessary to ensure vegetable safety and assess potential risks.

X-ray fluorescence (XRF) spectroscopy, a qualitative and quantitative technique for the simultaneous analysis of multiple elements, has the advantage of non-destructive analysis and is well suited for the identification and analysis of the essential and toxic elements in plants (Singh *et al.*, 2022 ▸), foods (Li *et al.*, 2022 ▸) and biological samples (Figueroa *et al.*, 2022 ▸). The distribution of elements in plants (Porfido *et al.*, 2023 ▸), such as in roots (Zandi *et al.*, 2023 ▸) and leaves (Nayak *et al.*, 2011 ▸), can be mapped using µ-XRF and has been widely used in agricultural, forestry and environmental research (Marguí *et al.*, 2022 ▸).

It is difficult to measure the original form of the element because the form of the element is always changed during sample processing. Therefore, avoiding chemical changes during sample pretreatment and analysis is key to obtaining the real elemental speciation. X-ray absorption near-edge structure spectroscopy (XANES) (Ketenoglu, 2022 ▸) is considered a potent technique for identifying the forms of low-content elements since it can be used for *in situ* analysis and to obtain information about the local structure of chosen elements in plants without extraction and other separation procedures (Buzanich, 2022 ▸). There are two types of application of XANES to plants. One focuses on the location and speciation of metals in hyperaccumulators (Pons *et al.*, 2021 ▸; Matzen *et al.*, 2022 ▸), and the other is dedicated to investigating HMs in food crops and vegetables (Jin *et al.*, 2022 ▸; Kunene *et al.*, 2021 ▸; Abbasi *et al.*, 2022 ▸; Brier *et al.*, 2016 ▸; Ogunkunle *et al.*, 2019 ▸). For instance, element distribution and Pb speciation in vegetable and grain seeds during germination have been studied using XANES and XRF. Luo *et al.* (2019 ▸) discovered that the distribution of elements within seeds was element-specific, and that distinct Pb complexes were formed in various growth periods and tissues. But what about the speciation and distribution features of HMs in leaf vegetable mature plants?

The objective of our investigation was to compare the Pb and interesting elements distribution features and identify the relevant Pb species in leeks – a popular vegetable with strong adaptability, cold and heat resistance, and widely cultivated in the north and south of China.

Understanding the migration accumulation pattern of Pb and providing a theoretical foundation for preventing Pb accumulation would facilitate the investigation of the migration capacity of Pb in leeks and its possible health concern.

## 2. Materials and methods

### 2.1. Leek cultivation

Leeks and soil were collected from a vegetable garden around the Qixiashan Pb–Zn mine in Nanjing, East China’s Jiangsu Province. Whole leeks were shovelled out along with the surrounding soil, transplanted into a pot, and brought back to the laboratory, before being placed outside on a windowsill to grow under natural light and temperature. Leeks are fine-leaved and white-rooted (*Allium tenuissimum L.*). To obtain better XANES maps, the leeks were irrigated with 500 mg L^−1^ PbNO_3_ solutions every five days and harvested after three weeks. The concentration of HMs in cultured soil was determined using pressed powder pellets via XRF spectrometry, and the contents of the HMs, shown in Table 1 ▸, were consistent with the range of HMs contents in the vegetable soil (Chu & Luo, 2010 ▸).

### 2.2. µ-XRF mapping

We selected the strongest leek plant, brushed away the soil around the leek root, and rinsed the entire plant several times with ultra-pure water. A 1.5 mm section of the main root was transected using a brain sectioning mold and mounted on a sample holder. The section was about 2 mm in diameter and about 1 cm from the stem.

The distributions of Pb and related elements in leek root were studied at an incident energy of 20 keV on the 15U1 beamline at the Shanghai Synchrotron Radiation Facility (Shanghai, China). The scanning spot size was 25 µm × 25 µm and the dwell time was 1.2 s. The root section was mounted on the *x*–*y* translation platform, and the displacement was shifted according to Δ*x* = 25 µm, Δ*y* = 25 µm. It was 80 steps in the *Y* direction and 74 steps in the *X* direction. Thus, images of 2000 µm and 1850 µm in length and width, respectively, were obtained. The fluorescence yield was measured with a seven-element germanium (Ge) solid-state detector and normalized by *I*_0_ and the dwell time. *IGOR Pro 6.03A* (https://www.wavemetrics.com/products/igorpro) was used for µ-XRF spectra analysis.

### 2.3. XANES

The hotspots on µ-XRF maps of leek root were selected for Pb XANES analysis. The leek leaf sample was chosen from the same plant, trimmed to 1.0 cm long, folded into two layers, and adhered to the holder with sulfur-free tape. The XANES spectra of leek samples were obtained on the 14W1 beamline at the Shanghai Synchrotron Radiation Facility (SSRF, Shanghai, China), after the µ-XRF mapping study. The XANES reference experiments were conducted on both the SSRF 14W1 beamline and the 1W1B beamline at the Beijing Synchrotron Radiation Facility (BSRF, Beijing, China). The first maximum of the first-order derivative of the reference lead foil spectrum was calibrated and the Pb *L*_III_-edge was set to 13035 eV. All leek samples had low concentrations – therefore measurements were made using a defocusing beam size of 100 µm × 100 µm, a seven-element Si (Li) solid-state detector in fluorescence mode and a germanium (Ge) filter if necessary. However, 13 powder control chemicals including inorganic lead, which is common in the environment, and organic lead, which binds to different ligands, were identified in transmission mode. All measurements were carried out in a room-temperature air environment. XANES spectra were measured in the energy range 12985–13155 eV with an equidistant energy step size of 0.5 eV. The first-order function and quadratic polynomial were used to normalize the pre-edge and post-edge regions of the obtained data, respectively, and *ATHENA* (version 0.8.061) software was used for data processing (Ravel & Newville, 2005 ▸).

## 3. Results

### 3.1. Spatial distribution of Pb and other elements of interest in root cross-sections

**Figure 1:**
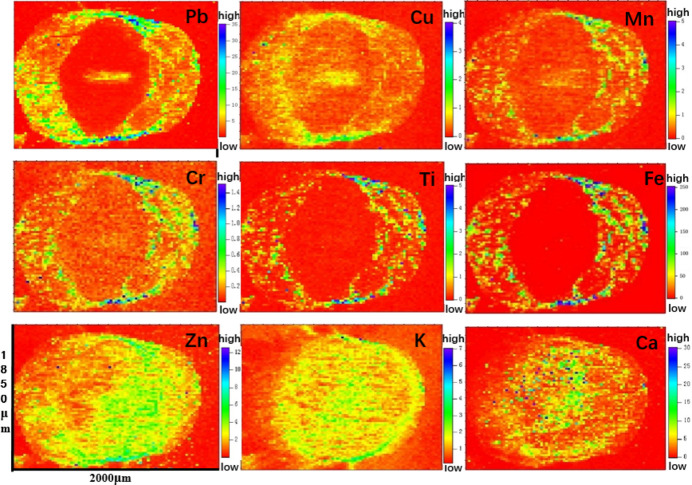
Cross-sectional µ-XRF of leek roots collected from the vegetable garden near the Pb–Zn mine and cultivated with 500 mg L^−1^ PbNO_3_ solutions for three weeks, showing spatial distributions of Pb, Cu, Mn, Cr, Ti, Fe, Zn, K and Ca. The color corresponds to the fluorescence count of each element. (All the values in the graph have to be multiplied by 10^−3^.)

When the plants were subjected to 500 mg L^−1^ lead stress, µ-XRF mapping revealed different distribution patterns of the elements in the root cross-section (Fig. 1 ▸). Pb, Cu, Mn, Cr, Ti and Fe were detected in the outer ring of the root cross-section with the most pronounced intensities, and high-intensity spots were observed in the epidermis. A lower intensity of Pb was trapped in the vascular bundle and was barely found in the endoderm. The distributions of Cu and Mn were similar to that of Pb but less intense than Pb. The distributions of Ti and Fe were extremely similar and were not detected in the endoderm and vascular bundle, except for in the outer ring and epidermis. The distributions of Pb, Cu, Mn, Cr, Ti and Fe were delimited in two or three parts of the root, while Zn, K and Ca were not obvious and were all distributed in the cross section of the root.

### 3.2. Pb XANES speciation

The morphology of an element in plants is crucial for their transformation, transport and function in the organism. The Pb species in the leek leaves and leek roots were determined by XANES, and its simulations were performed with 13 Pb reference compounds.

**Figure 2:**
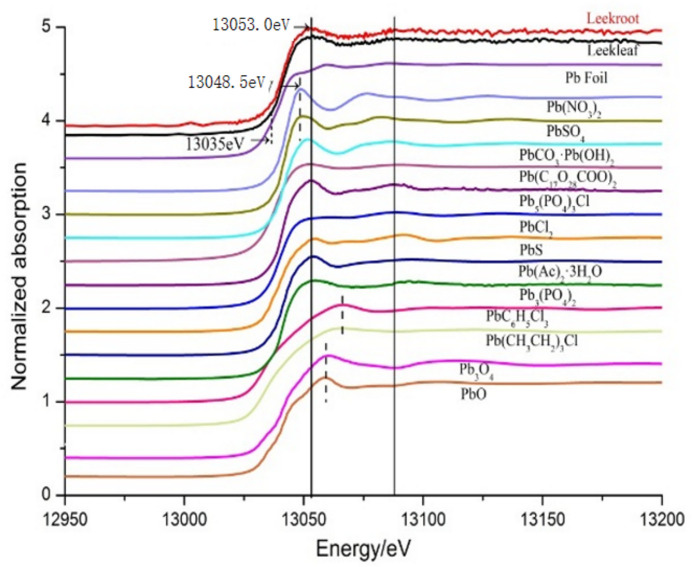
Pb *L*_III_-edge XANES spectra of leek root, leek leaf and standard references.

The Pb *L*_III_-edge XANES spectra of selected leek samples and the reference compounds are presented in Fig. 2 ▸. The XANES spectra of the Pb species in the leek leaf and leek root were very similar, in which the position of the white line of Pb was 13053.0 eV, an increase of 4.5 eV compared with 13048.5 eV for Pb nitrate. The displacement of the Pb *L*_III_-edge energy in leeks may indicate that leeks have the function of converting lead nitrate into other forms and storing them in cells or tissues.

The white-line energy position of Pb was roughly in the following order: Pb(NO_3_)_2_, PbSO_4_ < PbCO_3_·Pb(OH)_2_, Pb(C_17_H_34_COO)_2_, Pb_5_(PO_4_)_3_Cl, PbCl_2_, PbS, Pb(Ac)_2_·3H_2_O, Pb_3_(PO_4_)_2_ < Pb_3_O_4_, PbO, < PbC_6_H_5_Cl_3_, Pb(C_2_H_5_)_3_Cl. In the unknown samples, Pb always exists in different forms. Therefore, the XANES spectra of leek root and leek leaf were fitted with a linear combination of Pb standard spectra. Since the spectra were more similar to leeks, PbCO_3_·Pb(OH)_2_, Pb(C_17_H_35_COO)_2_, Pb_5_(PO_4_)_3_Cl, PbCl_2_, PbS, Pb_3_(PO_4_)_2_ and Pb(Ac)_2_·3H_2_O were selected for a linear combination fitting (LCF) calculations.

The presence and nature of probable components in the samples were verified using target transformation and principal component analysis (Gaur & Shrivastava, 2012 ▸). The LCF between unknown samples and standard samples was used to identify the species and weight of standards in the heterogeneous sample (Liu & Luo, 2019 ▸). The energy range was adjusted to −20 to +50 eV after *E*_0_ was originally set to 13035 eV. Included were the second apparent oscillation of the spectrum line and the ‘white line’. Under normalization conditions, the LCF can combine up to three standard spectra.

The fitting results and the fitting quality parameters are shown in Table 2 ▸. The normalized sum of the residuals was equal to the *R*-factor value (Niazi *et al.*, 2011 ▸). χ^2^ can be calculated by the sum of the squared differences between the data points and the model fit by the variance and degrees of freedom (Herndon *et al.*, 2014 ▸). The lower the *R*-factor, the smaller the fitting error and the more accurate the fitting result. This inference also applies to χ^2^ (Ravel & Newville, 2005 ▸).

**Figure 3:**
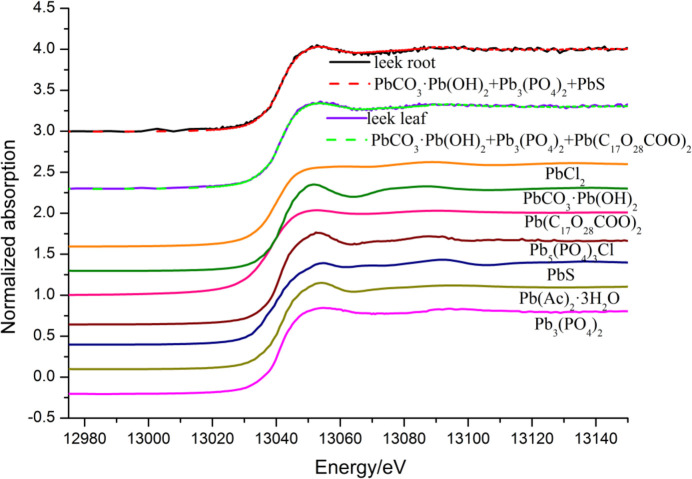
Pb *L*_III_-edge XANES spectra of leek samples and standard references were used for LCF. Dashed lines represent the results of LCF.

As shown in Fig. 3 ▸ and Table 1 ▸, the best fits for Pb in leek root were for lead phosphate [Pb_3_(PO4)_2_], basic lead carbonate [PbCO_3_·Pb(OH)_2_] and lead sulfide (PbS), which were about 54%, 27% and 19% by weight, respectively. The LCF of XANES spectra indicated that leek leaf Pb consisted mainly of Pb_3_(PO_4_)_2_ (68.0%), a small amount of PbCO_3_·Pb(OH)_2_ (20.9%) and minor levels of lead stearate [Pb(C_17_H_34_COO)_2_] (11.1%).

## 4. Discussion

### 4.1. Mechanisms of elemental interactions

The macronutrients in the soil can largely determine the behavior and intensity of interactions of other trace elements in the soil–plant system, such as Ca, P and Mg, *etc*., which can effectively antagonize the absorption of other trace elements (Ehlken & Kirchner, 2002 ▸). According to Roivainen *et al.* (2012 ▸), soil K, Mg, Mn, P and S levels were the most significant determinants impacting the transfer of the elements from the soil to the plant, and in most cases they had an inhibitory or competitive influence on other elements. In our study, K, Ca as well as Zn were distributed entirely in the cross section of the root, suggesting they played an essential role in the absorption of other elements.

Mn, Cr and Ti have similar physical and chemical properties to Fe, and they compete for the site of certain enzymes (Lyu *et al.*, 2017 ▸; Madejczyk & Ballatori, 2012 ▸; Pandey & Sharma, 2003 ▸; Rodrigues *et al.*, 2018 ▸). For example, Cr(III) replaces Fe(III) in heme proteins to reduce their activity (Pandey & Sharma, 2003 ▸). In tomato fruits, the Fe concentration rose whereas the K, Mg and Mn concentrations declined along with the concentrations of both individual and total amino acids (Zhang *et al.*, 2023 ▸). In the presence of limited iron availability, application of appropriate concentrations of Ti will induce iron regulatory transporter or yellow stripe 1 expression, thereby increasing iron uptake. If the concentration of Ti is too high, it will interfere with the biological functions of Fe, and lead to Ti poisoning (Lyu *et al.*, 2017 ▸). This could explain why Fe and Ti have such similar distributions in leek root.

According to studies by Salem *et al.* (2016 ▸), Fe accumulated in every part of the tomato plant whereas Pb, Co, Cr, Mn, Ti, Zn and Cu accumulated in the roots system of tomatoes, and only Pb was transported from roots to stems. However, in our study, in addition to Cr, Ti and Fe, Pb, Cu, Mn, Zn, K and Ca were present in the central cylinder and vascular tissues, suggesting that they can be transported to the above-ground parts. In *Brassica juncea*, the metal concentrations were Pb > Cu > Zn > Cd in roots and Zn > Cu > Cd > Pb in leaves (Kutrowska *et al.*, 2017 ▸), which suggested that, even though they can all be transported to the leaves, roots block Pb the most and allow Zn to pass first, and, in addition, the presence of Zn increases the accumulation of Pb and Zn. The same phenomenon of Pb and Cu was observed in cucumbers (An *et al.*, 2004 ▸), also demonstrating the poor mobility of Pb (Baycu *et al.*, 2006 ▸; Nicola *et al.*, 2003 ▸). However, other elements (Cd, Zn, Cu and Ca) in different combinations may increase the Pb accumulation factor and/or the mobility factor (An *et al.*, 2004 ▸; Kutrowska *et al.*, 2017 ▸; Sun & Luo, 2014 ▸). Therefore, both single high Pb levels and combined contamination of Pb with other elements may increase the risk of Pb overload in leeks (Sun & Luo, 2014 ▸).

### 4.2. Pb speciation in leeks

When Pb builds up in plants, several stable Pb compounds are produced for detoxification. The result of our study provided direct evidence for the conversion of Pb^2+^ in leek root and leek leaf mainly to Pb_3_(PO_4_)_2_ and PbCO_3_ with a proportion of more than 80%. Pb phosphate compounds (or pyromorphite) were also detected in various plant organs, including corn root (Luo *et al.*, 2019 ▸; Sun & Luo, 2018 ▸), waterlogged dropwort (*Oenanthe javanica DC*) (Liu & Luo, 2019 ▸), Indian mustard (Yang *et al.*, 2021 ▸) and roots of tea plant (Duan *et al.*, 2014 ▸). Lead carbonate was also found in *Phaseolus vulgeris* (Sarret *et al.*, 2001 ▸) and lettuce leaves (Schreck *et al.*, 2014 ▸). By physiologically converting Pb nitrate in the starting material into insoluble Pb carbonate and pyromorphite, the cell wall significantly reduces the toxicity of Pb (Sun & Luo, 2018 ▸; Yang *et al.*, 2021 ▸).

### 4.3. The tolerance against HMs

**Figure 4:**
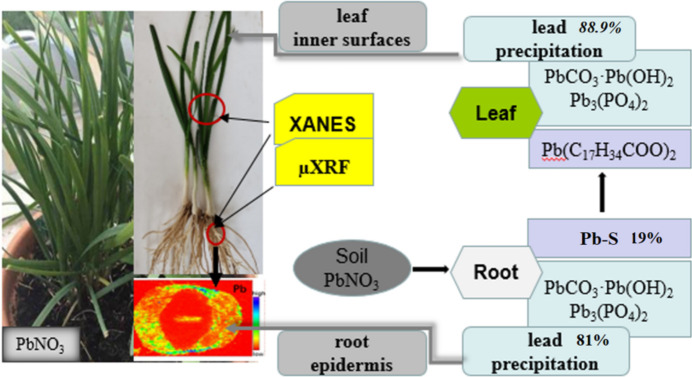
Absorption and resistance mechanism of Pb in leeks.

Heavy metal ions are mainly taken up by the root system and retained there, thus their translocation to above-ground parts is restricted (Salt *et al.*, 1995 ▸). In our previous study, the ratio of leek root Pb concentration to leaf Pb concentration was about 2.5–4.5 (Sun & Luo, 2014 ▸), which corresponded to 22%–40% of Pb being transported to the above-ground portion. The result is coincident with our present study that the stable Pb species Pb_3_(PO_4_)_2_ and PbCO_3_ account for 81%; in addition, Pb–S compounds are likely to be transferred to leek leaves with a proportion of 19%. Peralta-Videa *et al.* (2009 ▸) showed that Pb was concentrated in the phloem tissues of hydro­ponic honey mesquite (*Prosopis sp.*) when treated with high concentrations of Pb (more than 50 mg L^−1^), which suggested that Pb moved through the xylem to the leaves and returned to the plant body through the phloem. It is consistent with the µ-XRF results in this study that Pb content was higher in the vascular bundle, which contains two parts – xylem and phloem. Bovenkamp *et al.* (2013 ▸) and Peralta-Videa *et al.* (2009 ▸) suggested that, because precipitation is the most likely form of lead compounds, Pb is first adsorbed to the outer surface of the root and then attached to different surfaces within the plants in the form of phosphate or carbonate. This may explain why precipitated lead in leek leaves remains as high as 88.9%. In other words, the amount of lead that could cause damage to leaf cells is very low, less than 10%. The absorption and resistance mechanism of Pb in leeks is shown in Fig. 4 ▸.

## 5. Conclusions

The distribution of Pb, Cu, Mn, Cr, Ti, Fe, Zn, K and Ca in leek roots and the chemical forms of Pb were studied by a combination of µ-XRF and XANES techniques.

Pb, Cu, Mn, Cr, Ti and Fe were mainly distributed on the root epidermis of leek, whereas Zn, K and Ca were distributed throughout the root cross-section. The spatial distribution of elements may indicate possible synergistic or antagonistic interactions between potentially toxic elements, thus additional plant species, elements, environmental conditions and other types of contaminants, including organic pollutants, could be investigated to explore contaminant interactions and to assess environmental and health risks.

The present study shows that leek roots can physiologically convert about 80% of environmental lead into insoluble lead phosphate and lead carbonate. The formation of a large amount of lead precipitation and immobilization on the root epidermis and other inner surfaces is an important mechanism of Pb detoxification.

However, seven of the 13 standard samples were selected for LCF because their spectra were very similar to the spectra of the leek samples. In fact, the crystal structure and the speciation of the real samples are more complex. So we will purchase or synthesize more lead compounds as standard substances and try to fit samples with more standards to find more likely forms of lead in plants.

## Figures and Tables

**Table 1:** Contents of heavy metals in cultured soil

Element	Pb	As	Cd	Cu	Zn	Cr
Concentration (mg kg^−1^)	787.24	68.63	4.37	69.81	823.15	78.34

**Table 2:** Linear combination fitting of leek samples

Sample	Standard	Weight (%)	*R*-factor	χ^2^
Leek root	Pb_3_(PO_4_)_2_	54.1	0.00036	0.0334
	PbCO_3_·Pb(OH)_2_	27.0		
	PbS	19.0		

Leek leaf	Pb_3_(PO_4_)_2_	68.0	0.00015	0.0144
	PbCO_3_·Pb(OH)_2_	20.9		
	Pb(C_17_H_34_COO)_2_	11.1		
